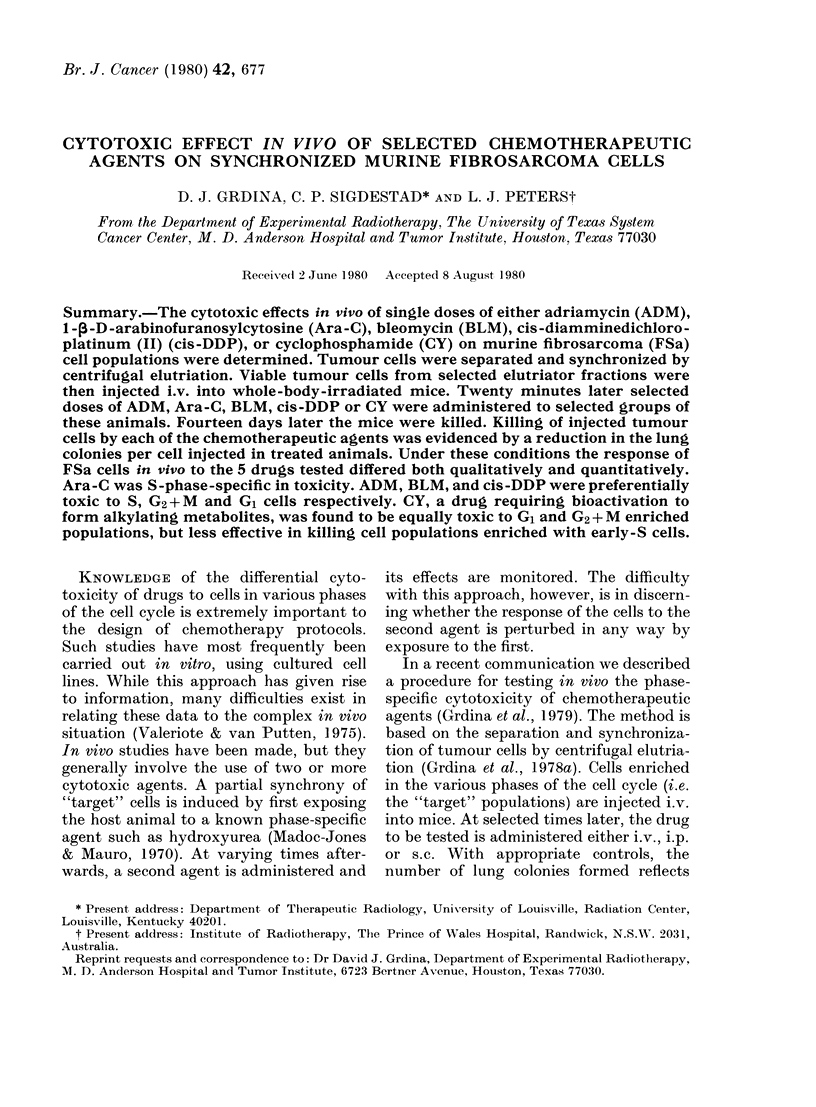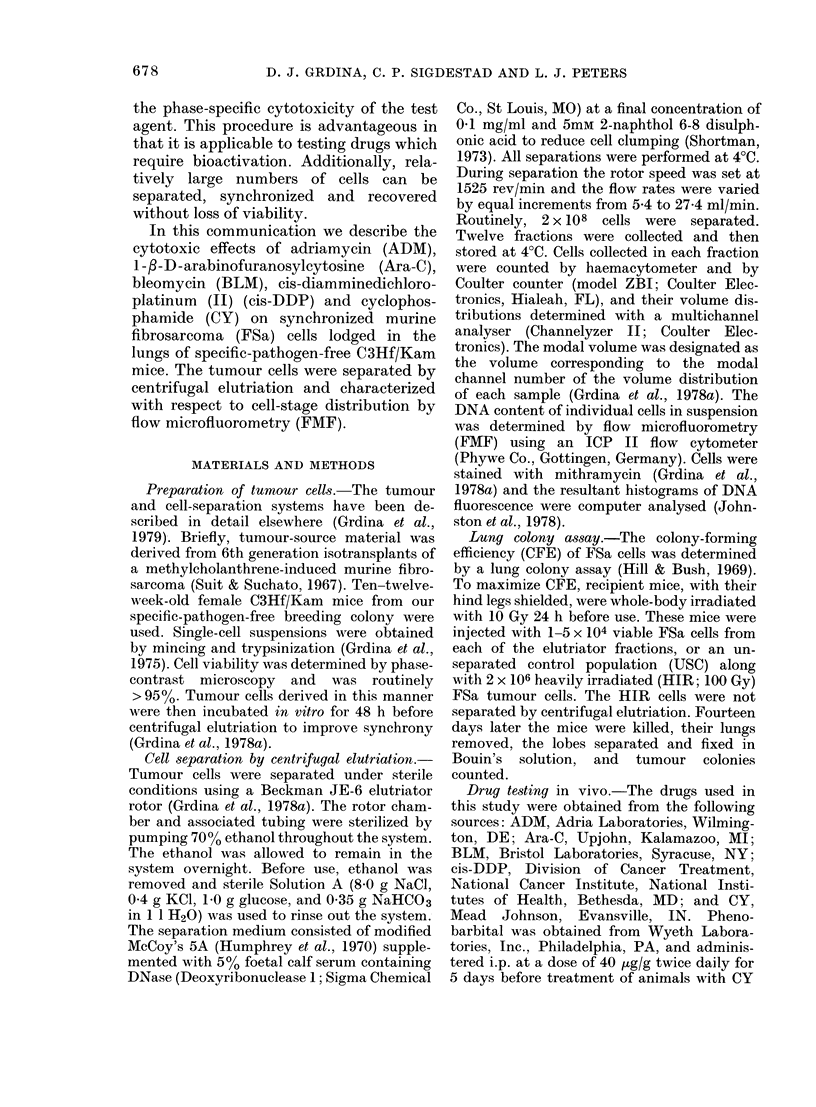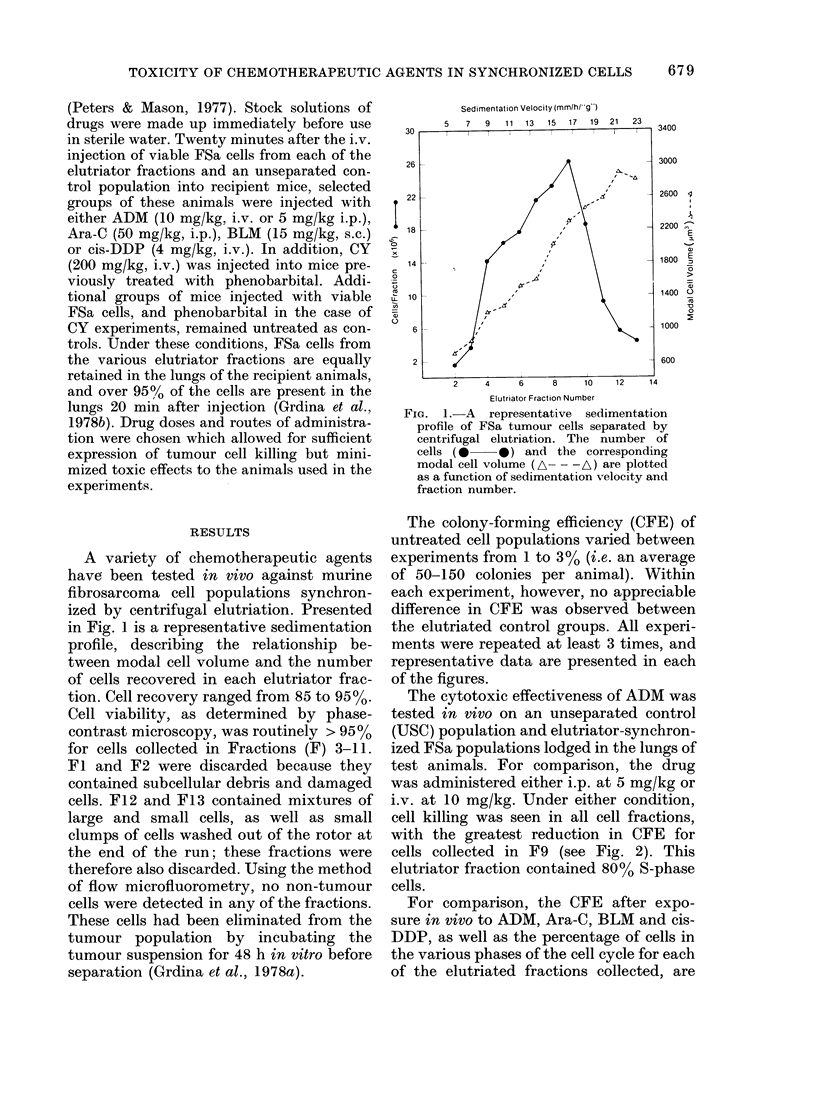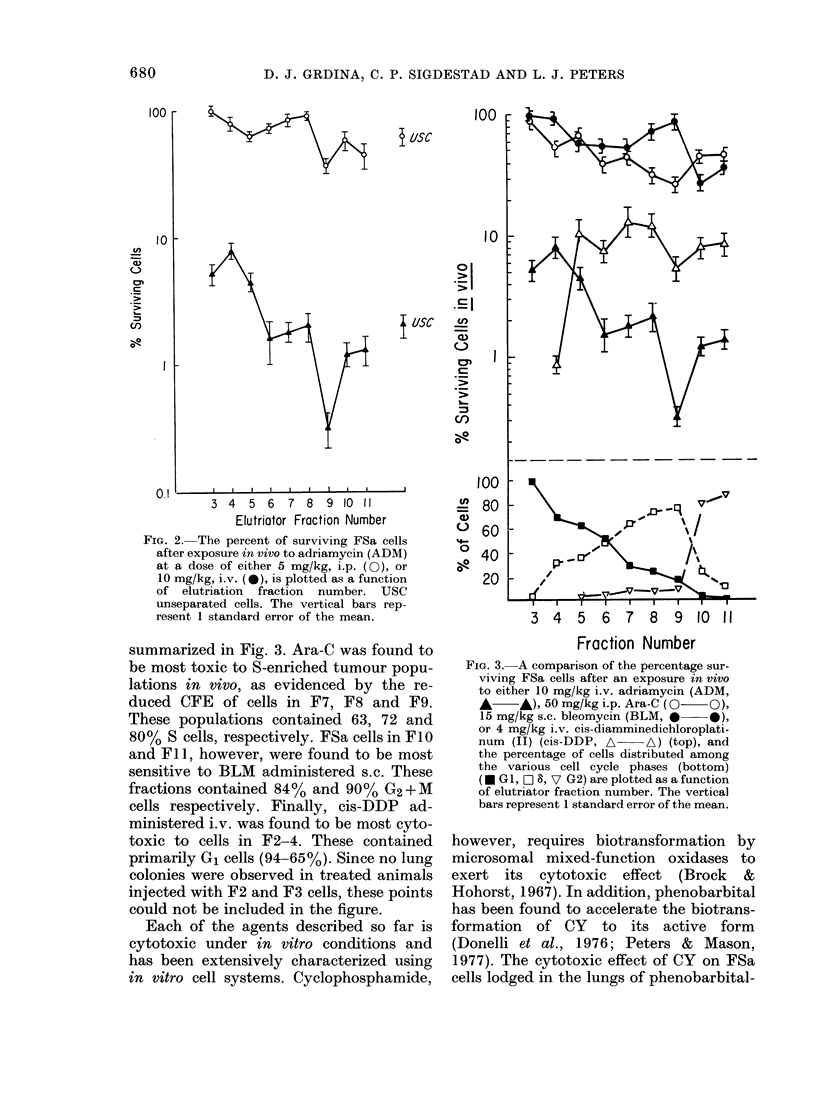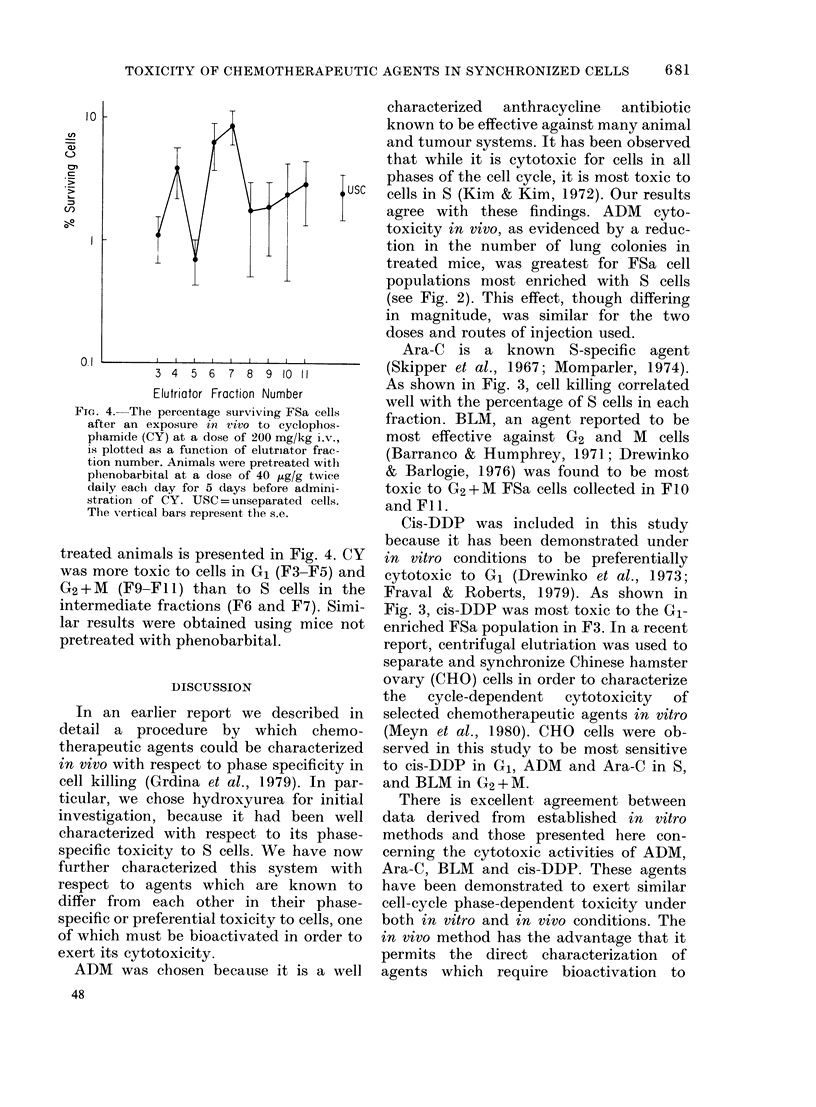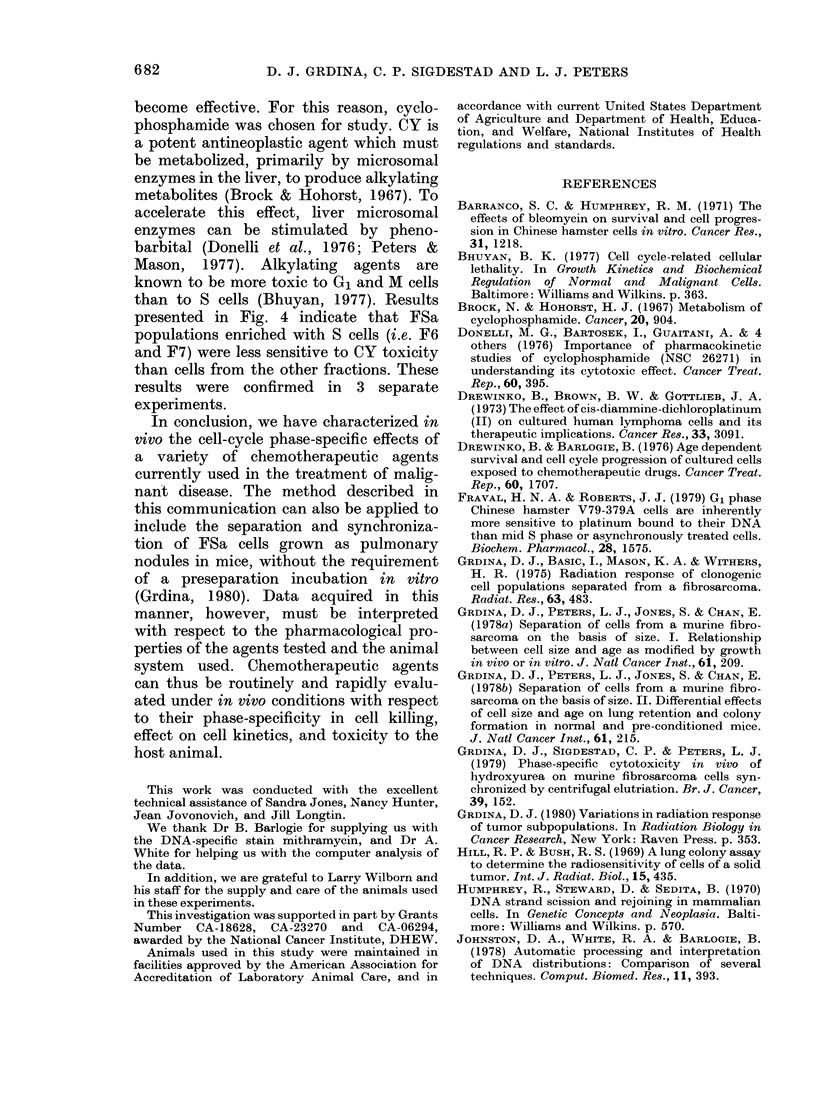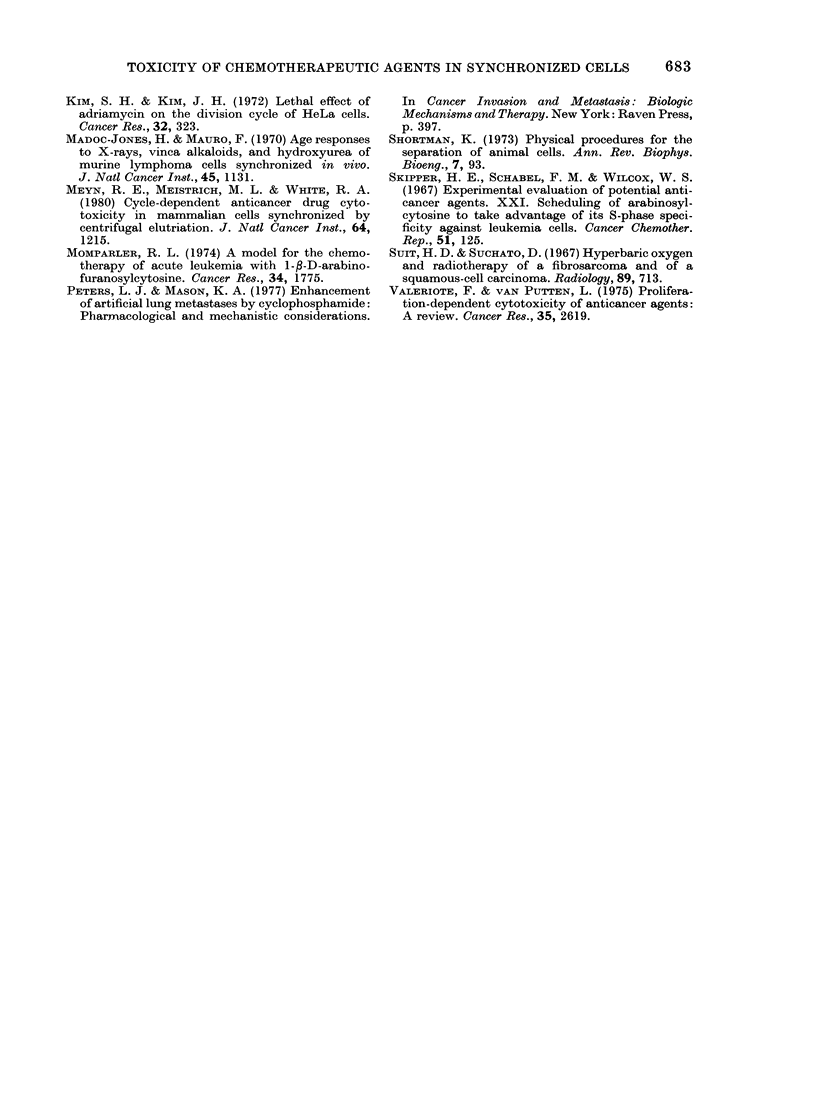# Cytotoxic effect in vivo of selected chemotherapeutic agents on synchronized murine fibrosarcoma cells.

**DOI:** 10.1038/bjc.1980.301

**Published:** 1980-11

**Authors:** D. J. Grdina, C. P. Sigdestad, L. J. Peters

## Abstract

The cytotoxic effects in vivo of single doses of either adriamycin (ADM), 1-beta-D-arabinofuranosylcytosine (Ara-C), bleomycin (BLM), cis-diamminedichloroplatinum (II) (cis-DDP), or cyclophosphamide (CY) on murine fibrosarcoma (FSa) cell populations were determined. Tumour cells were separated and synchronized by centrifugal elutriation. Viable tumour cells from selected elutriator fractions were then injected i.v. into whole-body-irradiated mice. Twenty minutes later selected doses of ADM, Ara-C, BLM, cis-DDP or CY were administered to selected groups of these animals. Fourteen days later the mice were killed. Killing of injected tumour cells by each of the chemotherapeutic agents was evidenced by a reduction in the lung cells by each of the chemotherapeutic agents was evidenced by a reduction in the lung colonies per cell injected in treated animals. Under these conditions the response of FSa cells in vivo to the 5 drugs tested differed both qualitatively and quantitatively. Ara-C was S-phase-specific in toxicity. ADM, BLM, and cis-DDP were preferentially toxic to S, G2+M and G1 cells respectively. CY, a drug requiring bioactivation to form alkylating metabolites, was found to be equally toxic to G1 and G2+M enriched populations, but less effective in killing cell populations enriched with early-S cells.


*Br. J. Cancer* (1980) **42,** 677

### CYTOTOXIC EFFECT *IN VIVO* OF SELECTED CHEMOTHERAPEUTIC AGENTS ON SYNCHRONIZED MURINE FIBROSARCOMA CELLS

D. J. GRDINA, C. P. SIGDESTAD* AND L. J. PETERS†

*From the Department of Experimental Radiotherapy, The University of Texas System Cancer Center, M. D. Anderson Hospital and Tumor Institute, Houston, Texas* 77030

Received 2 June 1980 Accepted 8 August 1980

**Summary.—The cytotoxic effects *in vivo* of single doses of either adriamycin (ADM), 1-β-D-arabinofuranosylcytosine (Ara-C), bleomycin (BLM), cis-diamminedichloroplatinum (II) (cis-DDP), or cyclophosphamide (CY) on murine fibrosarcoma (FSa) cell populations were determined. Tumour cells were separated and synchronized by centrifugal elutriation. Viable tumour cells from selected elutriator fractions were then injected i.v. into whole-body-irradiated mice. Twenty minutes later selected doses of ADM, Ara-C, BLM, cis-DDP or CY were administered to selected groups of these animals. Fourteen days later the mice were killed. Killing of injected tumour cells by each of the chemotherapeutic agents was evidenced by a reduction in the lung colonies per cell injected in treated animals. Under these conditions the response of FSa cells *in vivo* to the 5 drugs tested differed both qualitatively and quantitatively. Ara-C was S-phase-specific in toxicity. ADM, BLM, and cis-DDP were preferentially toxic to S, $G_2$+M and $G_1$ cells respectively. CY, a drug requiring bioactivation to form alkylating metabolites, was found to be equally toxic to $G_1$ and $G_2$+M enriched populations, but less effective in killing cell populations enriched with early-S cells.**

KNOWLEDGE of the differential cytotoxicity of drugs to cells in various phases of the cell cycle is extremely important to the design of chemotherapy protocols. Such studies have most frequently been carried out *in vitro*, using cultured cell lines. While this approach has given rise to information, many difficulties exist in relating these data to the complex *in vivo* situation (Valeriote & van Putten, 1975). *In vivo* studies have been made, but they generally involve the use of two or more cytotoxic agents. A partial synchrony of "target" cells is induced by first exposing the host animal to a known phase-specific agent such as hydroxyurea (Madoc-Jones & Mauro, 1970). At varying times afterwards, a second agent is administered and its effects are monitored. The difficulty with this approach, however, is in discerning whether the response of the cells to the second agent is perturbed in any way by exposure to the first.

In a recent communication we described a procedure for testing *in vivo* the phase-specific cytotoxicity of chemotherapeutic agents (Grdina *et al.*, 1979). The method is based on the separation and synchronization of tumour cells by centrifugal elutriation (Grdina *et al.*, 1978*a*). Cells enriched in the various phases of the cell cycle (*i.e.* the "target" populations) are injected i.v. into mice. At selected times later, the drug to be tested is administered either i.v., i.p. or s.c. With appropriate controls, the number of lung colonies formed reflects

* Present address: Department of Therapeutic Radiology, University of Louisville, Radiation Center, Louisville, Kentucky 40201.

† Present address: Institute of Radiotherapy, The Prince of Wales Hospital, Randwick, N.S.W. 2031, Australia.

Reprint requests and correspondence to: Dr David J. Grdina, Department of Experimental Radiotherapy, M. D. Anderson Hospital and Tumor Institute, 6723 Bertner Avenue, Houston, Texas 77030.

the phase-specific cytotoxicity of the test agent. This procedure is advantageous in that it is applicable to testing drugs which require bioactivation. Additionally, relatively large numbers of cells can be separated, synchronized and recovered without loss of viability.

In this communication we describe the cytotoxic effects of adriamycin (ADM), 1-β-D-arabinofuranosylcytosine (Ara-C), bleomycin (BLM), cis-diamminedichloroplatinum (II) (cis-DDP) and cyclophosphamide (CY) on synchronized murine fibrosarcoma (FSa) cells lodged in the lungs of specific-pathogen-free C3Hf/Kam mice. The tumour cells were separated by centrifugal elutriation and characterized with respect to cell-stage distribution by flow microfluorometry (FMF).

### MATERIALS AND METHODS

*Preparation of tumour cells.*—The tumour and cell-separation systems have been described in detail elsewhere (Grdina *et al.*, 1979). Briefly, tumour-source material was derived from 6th generation isotransplants of a methylcholanthrene-induced murine fibrosarcoma (Suit & Suchato, 1967). Ten–twelve-week-old female C3Hf/Kam mice from our specific-pathogen-free breeding colony were used. Single-cell suspensions were obtained by mincing and trypsinization (Grdina *et al.*, 1975). Cell viability was determined by phase-contrast microscopy and was routinely >95%. Tumour cells derived in this manner were then incubated *in vitro* for 48 h before centrifugal elutriation to improve synchrony (Grdina *et al.*, 1978*a*).

*Cell separation by centrifugal elutriation.*—Tumour cells were separated under sterile conditions using a Beckman JE-6 elutriator rotor (Grdina *et al.*, 1978*a*). The rotor chamber and associated tubing were sterilized by pumping 70% ethanol throughout the system. The ethanol was allowed to remain in the system overnight. Before use, ethanol was removed and sterile Solution A (8·0 g NaCl, 0·4 g KCl, 1·0 g glucose, and 0·35 g $NaHCO_3$ in 1 l $H_2O$) was used to rinse out the system. The separation medium consisted of modified McCoy's 5A (Humphrey *et al.*, 1970) supplemented with 5% foetal calf serum containing DNase (Deoxyribonuclease 1; Sigma Chemical Co., St Louis, MO) at a final concentration of 0·1 mg/ml and 5mM 2-naphthol 6-8 disulphonic acid to reduce cell clumping (Shortman, 1973). All separations were performed at 4°C. During separation the rotor speed was set at 1525 rev/min and the flow rates were varied by equal increments from 5·4 to 27·4 ml/min. Routinely, $2 \times 10^8$ cells were separated. Twelve fractions were collected and then stored at 4°C. Cells collected in each fraction were counted by haemacytometer and by Coulter counter (model ZBI; Coulter Electronics, Hialeah, FL), and their volume distributions determined with a multichannel analyser (Channelyzer II; Coulter Electronics). The modal volume was designated as the volume corresponding to the modal channel number of the volume distribution of each sample (Grdina *et al.*, 1978*a*). The DNA content of individual cells in suspension was determined by flow microfluorometry (FMF) using an ICP II flow cytometer (Phywe Co., Gottingen, Germany). Cells were stained with mithramycin (Grdina *et al.*, 1978*a*) and the resultant histograms of DNA fluorescence were computer analysed (Johnston *et al.*, 1978).

*Lung colony assay.*—The colony-forming efficiency (CFE) of FSa cells was determined by a lung colony assay (Hill & Bush, 1969). To maximize CFE, recipient mice, with their hind legs shielded, were whole-body irradiated with 10 Gy 24 h before use. These mice were injected with $1–5 \times 10^4$ viable FSa cells from each of the elutriator fractions, or an unseparated control population (USC) along with $2 \times 10^6$ heavily irradiated (HIR; 100 Gy) FSa tumour cells. The HIR cells were not separated by centrifugal elutriation. Fourteen days later the mice were killed, their lungs removed, the lobes separated and fixed in Bouin's solution, and tumour colonies counted.

*Drug testing* in vivo.—The drugs used in this study were obtained from the following sources: ADM, Adria Laboratories, Wilmington, DE; Ara-C, Upjohn, Kalamazoo, MI; BLM, Bristol Laboratories, Syracuse, NY; cis-DDP, Division of Cancer Treatment, National Cancer Institute, National Institutes of Health, Bethesda, MD; and CY, Mead Johnson, Evansville, IN. Phenobarbital was obtained from Wyeth Laboratories, Inc., Philadelphia, PA, and administered i.p. at a dose of 40 μg/g twice daily for 5 days before treatment of animals with CY

(Peters & Mason, 1977). Stock solutions of drugs were made up immediately before use in sterile water. Twenty minutes after the i.v. injection of viable FSa cells from each of the elutriator fractions and an unseparated control population into recipient mice, selected groups of these animals were injected with either ADM (10 mg/kg, i.v. or 5 mg/kg i.p.), Ara-C (50 mg/kg, i.p.), BLM (15 mg/kg, s.c.) or cis-DDP (4 mg/kg, i.v.). In addition, CY (200 mg/kg, i.v.) was injected into mice previously treated with phenobarbital. Additional groups of mice injected with viable FSa cells, and phenobarbital in the case of CY experiments, remained untreated as controls. Under these conditions, FSa cells from the various elutriator fractions are equally retained in the lungs of the recipient animals, and over 95% of the cells are present in the lungs 20 min after injection (Grdina *et al.*, 1978*b*). Drug doses and routes of administration were chosen which allowed for sufficient expression of tumour cell killing but minimized toxic effects to the animals used in the experiments.

FIG. 1.—A representative sedimentation profile of FSa tumour cells separated by centrifugal elutriation. The number of cells (●——●) and the corresponding modal cell volume (△- - -△) are plotted as a function of sedimentation velocity and fraction number.

### RESULTS

A variety of chemotherapeutic agents have been tested *in vivo* against murine fibrosarcoma cell populations synchronized by centrifugal elutriation. Presented in Fig. 1 is a representative sedimentation profile, describing the relationship between modal cell volume and the number of cells recovered in each elutriator fraction. Cell recovery ranged from 85 to 95%. Cell viability, as determined by phase-contrast microscopy, was routinely > 95% for cells collected in Fractions (F) 3–11. F1 and F2 were discarded because they contained subcellular debris and damaged cells. F12 and F13 contained mixtures of large and small cells, as well as small clumps of cells washed out of the rotor at the end of the run; these fractions were therefore also discarded. Using the method of flow microfluorometry, no non-tumour cells were detected in any of the fractions. These cells had been eliminated from the tumour population by incubating the tumour suspension for 48 h *in vitro* before separation (Grdina *et al.*, 1978*a*).

The colony-forming efficiency (CFE) of untreated cell populations varied between experiments from 1 to 3% (*i.e.* an average of 50–150 colonies per animal). Within each experiment, however, no appreciable difference in CFE was observed between the elutriated control groups. All experiments were repeated at least 3 times, and representative data are presented in each of the figures.

The cytotoxic effectiveness of ADM was tested *in vivo* on an unseparated control (USC) population and elutriator-synchronized FSa populations lodged in the lungs of test animals. For comparison, the drug was administered either i.p. at 5 mg/kg or i.v. at 10 mg/kg. Under either condition, cell killing was seen in all cell fractions, with the greatest reduction in CFE for cells collected in F9 (see Fig. 2). This elutriator fraction contained 80% S-phase cells.

For comparison, the CFE after exposure *in vivo* to ADM, Ara-C, BLM and cis-DDP, as well as the percentage of cells in the various phases of the cell cycle for each of the elutriated fractions collected, are

FIG. 2.—The percent of surviving FSa cells after exposure *in vivo* to adriamycin (ADM) at a dose of either 5 mg/kg, i.p. (○), or 10 mg/kg, i.v. (●), is plotted as a function of elutriation fraction number. USC unseparated cells. The vertical bars represent 1 standard error of the mean.

FIG. 3.—A comparison of the percentage surviving FSa cells after an exposure *in vivo* to either 10 mg/kg i.v. adriamycin (ADM, ▲——▲), 50 mg/kg i.p. Ara-C (○——○), 15 mg/kg s.c. bleomycin (BLM, ●——●), or 4 mg/kg i.v. cis-diamminedichloroplatinum (II) (cis-DDP, △——△) (top), and the percentage of cells distributed among the various cell cycle phases (bottom) (■ G1, □ S, ▽ G2) are plotted as a function of elutriator fraction number. The vertical bars represent 1 standard error of the mean.

summarized in Fig. 3. Ara-C was found to be most toxic to S-enriched tumour populations *in vivo*, as evidenced by the reduced CFE of cells in F7, F8 and F9. These populations contained 63, 72 and 80% S cells, respectively. FSa cells in F10 and F11, however, were found to be most sensitive to BLM administered s.c. These fractions contained 84% and 90% $G_2$+M cells respectively. Finally, cis-DDP administered i.v. was found to be most cytotoxic to cells in F2–4. These contained primarily $G_1$ cells (94–65%). Since no lung colonies were observed in treated animals injected with F2 and F3 cells, these points could not be included in the figure.

Each of the agents described so far is cytotoxic under *in vitro* conditions and has been extensively characterized using *in vitro* cell systems. Cyclophosphamide, however, requires biotransformation by microsomal mixed-function oxidases to exert its cytotoxic effect (Brock & Hohorst, 1967). In addition, phenobarbital has been found to accelerate the biotransformation of CY to its active form (Donelli *et al.*, 1976; Peters & Mason, 1977). The cytotoxic effect of CY on FSa cells lodged in the lungs of phenobarbital-

FIG. 4.—The percentage surviving FSa cells after an exposure *in vivo* to cyclophosphamide (CY) at a dose of 200 mg/kg i.v., is plotted as a function of elutriator fraction number. Animals were pretreated with phenobarbital at a dose of 40 $\mu$g/g twice daily each day for 5 days before administration of CY. USC = unseparated cells. The vertical bars represent the s.e.

treated animals is presented in Fig. 4. CY was more toxic to cells in $G_1$ (F3–F5) and $G_2$+M (F9–F11) than to S cells in the intermediate fractions (F6 and F7). Similar results were obtained using mice not pretreated with phenobarbital.

### DISCUSSION

In an earlier report we described in detail a procedure by which chemotherapeutic agents could be characterized *in vivo* with respect to phase specificity in cell killing (Grdina *et al.*, 1979). In particular, we chose hydroxyurea for initial investigation, because it had been well characterized with respect to its phase-specific toxicity to S cells. We have now further characterized this system with respect to agents which are known to differ from each other in their phase-specific or preferential toxicity to cells, one of which must be bioactivated in order to exert its cytotoxicity.

ADM was chosen because it is a well characterized anthracycline antibiotic known to be effective against many animal and tumour systems. It has been observed that while it is cytotoxic for cells in all phases of the cell cycle, it is most toxic to cells in S (Kim & Kim, 1972). Our results agree with these findings. ADM cytotoxicity *in vivo*, as evidenced by a reduction in the number of lung colonies in treated mice, was greatest for FSa cell populations most enriched with S cells (see Fig. 2). This effect, though differing in magnitude, was similar for the two doses and routes of injection used.

Ara-C is a known S-specific agent (Skipper *et al.*, 1967; Momparler, 1974). As shown in Fig. 3, cell killing correlated well with the percentage of S cells in each fraction. BLM, an agent reported to be most effective against $G_2$ and M cells (Barranco & Humphrey, 1971; Drewinko & Barlogie, 1976) was found to be most toxic to $G_2$+M FSa cells collected in F10 and F11.

Cis-DDP was included in this study because it has been demonstrated under *in vitro* conditions to be preferentially cytotoxic to $G_1$ (Drewinko *et al.*, 1973; Fraval & Roberts, 1979). As shown in Fig. 3, cis-DDP was most toxic to the $G_1$-enriched FSa population in F3. In a recent report, centrifugal elutriation was used to separate and synchronize Chinese hamster ovary (CHO) cells in order to characterize the cycle-dependent cytotoxicity of selected chemotherapeutic agents *in vitro* (Meyn *et al.*, 1980). CHO cells were observed in this study to be most sensitive to cis-DDP in $G_1$, ADM and Ara-C in S, and BLM in $G_2$+M.

There is excellent agreement between data derived from established *in vitro* methods and those presented here concerning the cytotoxic activities of ADM, Ara-C, BLM and cis-DDP. These agents have been demonstrated to exert similar cell-cycle phase-dependent toxicity under both *in vitro* and *in vivo* conditions. The *in vivo* method has the advantage that it permits the direct characterization of agents which require bioactivation to become effective. For this reason, cyclophosphamide was chosen for study. CY is a potent antineoplastic agent which must be metabolized, primarily by microsomal enzymes in the liver, to produce alkylating metabolites (Brock & Hohorst, 1967). To accelerate this effect, liver microsomal enzymes can be stimulated by phenobarbital (Donelli *et al.*, 1976; Peters & Mason, 1977). Alkylating agents are known to be more toxic to $G_1$ and M cells than to S cells (Bhuyan, 1977). Results presented in Fig. 4 indicate that FSa populations enriched with S cells (*i.e.* F6 and F7) were less sensitive to CY toxicity than cells from the other fractions. These results were confirmed in 3 separate experiments.

In conclusion, we have characterized *in vivo* the cell-cycle phase-specific effects of a variety of chemotherapeutic agents currently used in the treatment of malignant disease. The method described in this communication can also be applied to include the separation and synchronization of FSa cells grown as pulmonary nodules in mice, without the requirement of a preseparation incubation *in vitro* (Grdina, 1980). Data acquired in this manner, however, must be interpreted with respect to the pharmacological properties of the agents tested and the animal system used. Chemotherapeutic agents can thus be routinely and rapidly evaluated under *in vivo* conditions with respect to their phase-specificity in cell killing, effect on cell kinetics, and toxicity to the host animal.

This work was conducted with the excellent technical assistance of Sandra Jones, Nancy Hunter, Jean Jovonovich, and Jill Longtin.

We thank Dr B. Barlogie for supplying us with the DNA-specific stain mithramycin, and Dr A. White for helping us with the computer analysis of the data.

In addition, we are grateful to Larry Wilborn and his staff for the supply and care of the animals used in these experiments.

This investigation was supported in part by Grants Number CA-18628, CA-23270 and CA-06294, awarded by the National Cancer Institute, DHEW.

Animals used in this study were maintained in facilities approved by the American Association for Accreditation of Laboratory Animal Care, and in accordance with current United States Department of Agriculture and Department of Health, Education, and Welfare, National Institutes of Health regulations and standards.

### REFERENCES

Barranco, S. C. & Humphrey, R. M. (1971) The effects of bleomycin on survival and cell progression in Chinese hamster cells *in vitro*. *Cancer Res.*, **31**, 1218.

Bhuyan, B. K. (1977) Cell cycle-related cellular lethality. In *Growth Kinetics and Biochemical Regulation of Normal and Malignant Cells*. Baltimore: Williams and Wilkins. p. 363.

Brock, N. & Hohorst, H. J. (1967) Metabolism of cyclophosphamide. *Cancer*, **20**, 904.

Donelli, M. G., Bartosek, I., Guaitani, A. & 4 others (1976) Importance of pharmacokinetic studies of cyclophosphamide (NSC 26271) in understanding its cytotoxic effect. *Cancer Treat. Rep.*, **60**, 395.

Drewinko, B., Brown, B. W. & Gottlieb, J. A. (1973) The effect of cis-diammine-dichloroplatinum (II) on cultured human lymphoma cells and its therapeutic implications. *Cancer Res.*, **33**, 3091.

Drewinko, B. & Barlogie, B. (1976) Age dependent survival and cell cycle progression of cultured cells exposed to chemotherapeutic drugs. *Cancer Treat. Rep.*, **60**, 1707.

Fraval, H. N. A. & Roberts, J. J. (1979) $G_1$ phase Chinese hamster V79-379A cells are inherently more sensitive to platinum bound to their DNA than mid S phase or asynchronously treated cells. *Biochem. Pharmacol.*, **28**, 1575.

Grdina, D. J., Basic, I., Mason, K. A. & Withers, H. R. (1975) Radiation response of clonogenic cell populations separated from a fibrosarcoma. *Radiat. Res.*, **63**, 483.

Grdina, D. J., Peters, L. J., Jones, S. & Chan, E. (1978*a*) Separation of cells from a murine fibrosarcoma on the basis of size. I. Relationship between cell size and age as modified by growth *in vivo* or *in vitro*. *J. Natl Cancer Inst.*, **61**, 209.

Grdina, D. J., Peters, L. J., Jones, S. & Chan, E. (1978*b*) Separation of cells from a murine fibrosarcoma on the basis of size. II. Differential effects of cell size and age on lung retention and colony formation in normal and pre-conditioned mice. *J. Natl Cancer Inst.*, **61**, 215.

Grdina, D. J., Sigdestad, C. P. & Peters, L. J. (1979) Phase-specific cytotoxicity *in vivo* of hydroxyurea on murine fibrosarcoma cells synchronized by centrifugal elutriation. *Br. J. Cancer*, **39**, 152.

Grdina, D. J. (1980) Variations in radiation response of tumor subpopulations. In *Radiation Biology in Cancer Research*, New York: Raven Press. p. 353.

Hill, R. P. & Bush, R. S. (1969) A lung colony assay to determine the radiosensitivity of cells of a solid tumor. *Int. J. Radiat. Biol.*, **15**, 435.

Humphrey, R., Steward, D. & Sedita, B. (1970) DNA strand scission and rejoining in mammalian cells. In *Genetic Concepts and Neoplasia*. Baltimore: Williams and Wilkins. p. 570.

Johnston, D. A., White, R. A. & Barlogie, B. (1978) Automatic processing and interpretation of DNA distributions: Comparison of several techniques. *Comput. Biomed. Res.*, **11**, 393.

KIM, S. H. & KIM, J. H. (1972) Lethal effect of adriamycin on the division cycle of HeLa cells. *Cancer Res.*, **32**, 323.

MADOC-JONES, H. & MAURO, F. (1970) Age responses to X-rays, vinca alkaloids, and hydroxyurea of murine lymphoma cells synchronized *in vivo*. *J. Natl Cancer Inst.*, **45**, 1131.

MEYN, R. E., MEISTRICH, M. L. & WHITE, R. A. (1980) Cycle-dependent anticancer drug cytotoxicity in mammalian cells synchronized by centrifugal elutriation. *J. Natl Cancer Inst.*, **64**, 1215.

MOMPARLER, R. L. (1974) A model for the chemotherapy of acute leukemia with 1-β-D-arabinofuranosylcytosine. *Cancer Res.*, **34**, 1775.

PETERS, L. J. & MASON, K. A. (1977) Enhancement of artificial lung metastases by cyclophosphamide: Pharmacological and mechanistic considerations. In *Cancer Invasion and Metastasis: Biologic Mechanisms and Therapy*. New York: Raven Press, p. 397.

SHORTMAN, K. (1973) Physical procedures for the separation of animal cells. *Ann. Rev. Biophys. Bioeng.*, **7**, 93.

SKIPPER, H. E., SCHABEL, F. M. & WILCOX, W. S. (1967) Experimental evaluation of potential anticancer agents. XXI. Scheduling of arabinosylcytosine to take advantage of its S-phase specificity against leukemia cells. *Cancer Chemother. Rep.*, **51**, 125.

SUIT, H. D. & SUCHATO, D. (1967) Hyperbaric oxygen and radiotherapy of a fibrosarcoma and of a squamous-cell carcinoma. *Radiology*, **89**, 713.

VALERIOTE, F. & VAN PUTTEN, L. (1975) Proliferation-dependent cytotoxicity of anticancer agents: A review. *Cancer Res.*, **35**, 2619.